# Diethyl Ether Conversion to Ethene and Ethanol Catalyzed by Heteropoly Acids

**DOI:** 10.1021/acsomega.1c00958

**Published:** 2021-03-23

**Authors:** Rawan Al-Faze, Elena F. Kozhevnikova, Ivan V. Kozhevnikov

**Affiliations:** † Department of Chemistry, University of Liverpool, Liverpool L69 7ZD, U.K.; ‡ Department of Chemistry, Taibah University, P.O. Box 30002, Al-Madinah Al-Munawarah 41147, Saudi Arabia

## Abstract

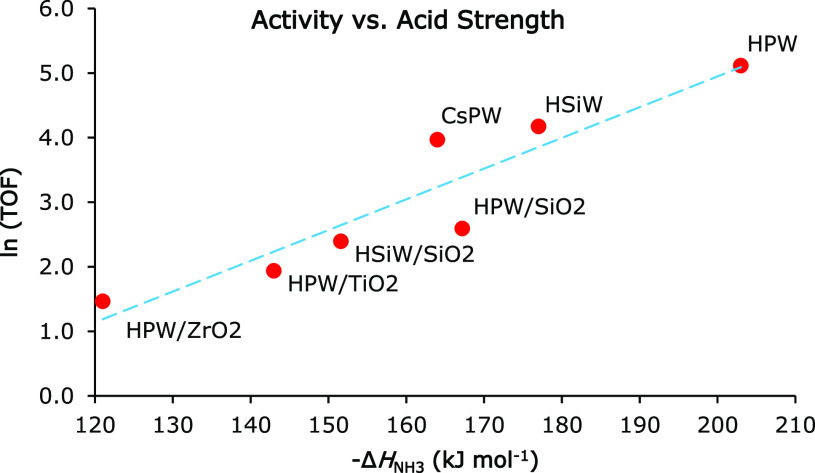

The conversion of diethyl ether (DEE) to ethene and ethanol was studied at a gas–solid interface over bulk and supported Brønsted solid acid catalysts based on tungsten Keggin heteropoly acids (HPAs) at 130–250 °C and ambient pressure. The yield of ethene increased with increasing reaction temperature and reached 98% at 220–250 °C (WHSV = 2.2 h^–1^). The most active HPA catalysts were silica-supported H_3_PW_12_O_40_ and H_4_SiW_12_O_40_ and the bulk heteropoly salt Cs_2_._5_H_0_._5_PW_12_O_40_. The HPA catalysts outperformed zeolites HZSM-5 and USY reported elsewhere. A correlation between catalyst activity and catalyst acid strength was established, which indicates that Brønsted acid sites play an important role in DEE elimination over HPA catalysts. The results point to the reaction occurring through the consecutive reaction pathway: DEE → C_2_H_4_ + EtOH followed by EtOH → C_2_H_4_ + H_2_O, where ethene is both a primary product of DEE elimination and a secondary product via dehydration of the primary product EtOH. Evidence is provided that DEE elimination over bulk HPA and high-loaded HPA/SiO_2_ catalysts proceeds via the surface-type mechanism.

## 1 Introduction

The dehydration of EtOH to ethene (eq 1) and diethyl ether (DEE) (eq 2) is of interest for the production of ethene and DEE from renewable nonpetroleum resources.^1−3^ Ethene is widely used in the chemical industry,^1,2^ and DEE is a valuable chemical, aprotic solvent, anesthetic, and green fuel alternative.^4^
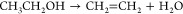
1
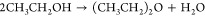
2
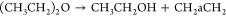
3

The dehydration of EtOH can be carried out in the gas or liquid phase in the presence of acid catalysts. As the catalysts, metal oxides, zeolites, and heteropoly acids (HPAs) are most often used.^5−17^ HPAs, having a stronger acidity, are more active in this reaction.^14−20^ DEE is a key intermediate in the ethanol-to-ethene dehydration.^10−13,16^ In the presence of an acid catalyst, DEE undergoes elimination to produce ethene and EtOH (eq 3); the latter, in turn, dehydrates to ethene (eq 1). The mechanism of ethene formation from EtOH dehydration is debated (ref (10) and references therein). Only DEE forms in EtOH dehydration at low temperatures, whereas ethene forms at higher temperatures, either directly from EtOH or via DEE cracking or both.^10^ Therefore, knowledge about the acid-catalyzed elimination of DEE can shed light on the mechanism of the ethanol-to-ethene dehydration. DEE elimination is also of interest in its own right to convert spent DEE into useful products such as ethene and ethanol.^11,13^

**Scheme 1:**
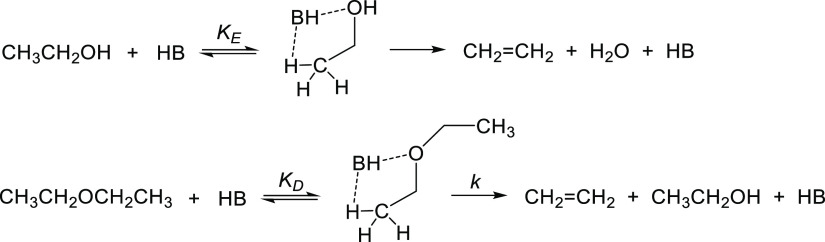
E2 Elimination of EtOH and DEE by a Pair of Acid (H^+^) and Base (B^–^) Sites

The ethanol-to-ethene dehydration is suggested to proceed through the bimolecular elimination mechanism E2. This mechanism involves simultaneous cleavage of C–O and C–H bonds in alcohol by a pair of acid and base catalyst sites (Scheme 1).^21^ From the general concept of heterolytic 1,2-elimination reactions,^22^ a similar E2 mechanism may be assumed for the acid-catalyzed DEE elimination to form ethene and EtOH (Scheme 1). The E2 mechanism has been suggested for the elimination of DEE on γ-Al_2_O_3_ from DFT modeling.^10^

HPAs of the Keggin structure are represented by the formula H_8–*x*_[X^*x*+^M_12_O_40_] (X^*x*+^ = P^5+^ or Si^4+^; M = W^6+^ or Mo^6+^). These HPAs are very strong Brønsted acids, stronger than the conventional solid acid catalysts such as metal oxides and zeolites. 12-Tungstophosphoric acid (H_3_PW_12_O_40_ (HPW)) and 12-tungstosilisic acid (H_4_SiW_12_O_40_ (HSiW)) are the strongest HPAs in the Keggin series. The acid properties of HPW and HSiW are well documented in the literature (refs (23−28) and references therein). These HPAs have been reported as efficient acid catalysts in a wide range of reactions,^23−28^ including the dehydration of EtOH.^14−20^ An HPA catalyst is used in Hummingbird technology for the dehydration of bioethanol to polymer-grade ethene.^29^

Several studies on DEE elimination over γ-alumina have been published,^10−13^ including kinetics^12^ and DFT analysis of the reaction mechanism.^10^ Phung and Basca^11^ have reported the elimination of DEE in the presence of zeolite and oxide catalysts at 150–450 °C, with catalyst activity decreasing in the order HZSM-5 > USY > γ-Al_2_O_3_ ≈ SiO_2_–Al_2_O_3_. The most active catalyst HZSM-5 gives 90% ethene selectivity at 100% DEE conversion at 350 °C; 10% of higher hydrocarbons is also formed.^11^ Little has been published, however, on the elimination of DEE over HPA catalysts so far, except for a brief report by Bokade and Yadav^16^ on DEE elimination over HPW supported on montmorillonite clay at 150–250 °C with a moderate ethene yield of 58% at 250 °C.

In this work, we investigate the elimination of DEE to ethene and ethanol in the presence of bulk and supported Brønsted solid acid catalysts based on tungsten Keggin HPAs (HPW and HSiW) at a gas–solid interface in a fixed-bed reactor. The properties of bulk and silica-supported HPA catalysts have been documented in very detail over the past several decades (refs (23−28) and references therein). The HPA catalysts that are studied in this work have been thoroughly characterized previously regarding their texture, structural integrity, and acid properties (acid strength and acid-site density).^20,30^ Our primary goal is to compare the activity and selectivity of HPA catalysts with those of the conventional catalysts such as zeolites and metal oxides. We also aim to establish a relationship between the turnover activity of HPA catalysts and their acid strength and to gain an insight into the reaction mechanism.

## 2 Experimental Section

### 2.1 Materials

HPA hydrates H_3_PW_12_O_40_ (99%, from Sigma-Aldrich) and H_4_SiW_12_O_40_ (99.9%, from Fluka) contained 20–28 H_2_O molecules per Keggin unit. DEE (>97%) was purchased from Fisher Scientific. Catalyst supports Aerosil 300 silica and P25 titania (anatase/rutile = 3:1) were obtained from Degussa. ZrO_2_ was prepared in-house, as described previously,^29^ and calcined at 400 °C in air for 4 h.

### 2.2 Catalyst Preparation

Supported HPA catalysts were prepared by wet impregnation of oxide supports (SiO_2_, TiO_2_, and ZrO_2_) with an aqueous HPA solution, as described elsewhere,^20,30^ and dried at 150 °C/1 Pa for 1.5 h. Caesium 12-tungstophosphate Cs_2.5_H_0.5_PW_12_O_40_ (CsPW) was prepared using the literature procedure^31^ by adding dropwise the required amount of aqueous solution of Cs_2_CO_3_ (0.47 M) to aqueous solution of H_3_PW_12_O_40_ (0.75 M) to afford CsPW as a white precipitate. The precipitate was aged in an aqueous mixture for 48 h at room temperature and dried at 150 °C/1 Pa for 1.5 h. The catalysts were ground and sieved to 45–180 μm particle size and kept in a desiccator over calcined silica gel. The HPA loading in the catalysts was determined from W analysis by ICP–OES (inductively coupled plasma optical emission spectroscopy). From TGA, the finished catalysts contained 5–7% of water; further drying was not practical because the reaction under study yielded H_2_O as a byproduct. Spent HPA catalysts after DEE conversion had a similar water content to the fresh catalysts. It should be noted that the catalysts which dried at higher temperatures (250 °C/1 Pa/1.5 h) exhibited a decrease in activity before reaching the steady state probably due to adsorption of water formed during reaction and catalyst coking. Information about the catalysts studied is given in Table 1.

**Table 1:** Catalyst Characterization

catalyst^a^	surface area (m^2^ g^–1^)^b^	pore volume (cm^3^ g^–1^)^c^	pore diameter (Å)^d^	–Δ*H*_o_ (kJ mol^–1^)^e^
ZrO_2_	95	0.1	31	
TiO_2_	44	0.1	90	
SiO_2_	283	1.2	164	
H_3_PW_12_O_40_ (HPW)	5.7	0.01	74	203
H_4_SiW_12_O_40_ (HSiW)	8.0	0.01	68	177
CsPW	130	0.1	27	164
14%HPW/ZrO_2_	69	0.1	29	121
15%HPW/TiO_2_	46	0.2	164	143
5.8%HPW/SiO_2_	265	1.1	161	137
11%HPW/SiO_2_	237	1.1	189	166
17%HPW/SiO_2_	226	0.9	158	167
26%HPW/SiO_2_	188	0.8	178	167
43%HPW/SiO_2_	150	0.6	150	179
57%HPW/SiO_2_	94	0.3	139	185
5.8%HSiW/SiO_2_	259	1.0	150	138
11%HSiW/SiO_2_	242	1.0	170	152
14%HSiW/SiO_2_	222	1.0	183	152
25%HSiW/SiO_2_	191	0.8	167	154
46%HSiW/SiO_2_	143	0.5	146	157
71%HSiW/SiO_2_	87	0.3	118	160

a HPA loading from ICP-OES analysis.

b BET surface area.

c Single-point total pore volume at *p*/*p*_o_ = 0.97.

d Average BET pore diameter.

e Initial enthalpy of NH_3_ adsorption extrapolated to zero NH_3_ coverage from ammonia adsorption microcalorimetry at 150 °C (±6 kJ mol^–1^).^20,30^

### 2.3 Techniques

The surface area and porosity of the catalysts were characterized by the BET method from nitrogen physisorption measured at −196 °C on a Micromeritics ASAP 2010 instrument. Before analysis, the samples were evacuated at 250 °C. Thermogravimetric analysis (TGA) was carried out on a Perkin Elmer TGA-7 instrument under a nitrogen atmosphere. The ICP-OES analysis of HPA catalysts was carried out on a Spectro Ciros ICP-OES instrument; catalyst samples were digested by boiling in 15% aqueous KOH.

### 2.4 Catalyst Testing

The conversion of DEE to ethene and ethanol was carried out at 150–250 °C under atmospheric pressure in a Pyrex fixed-bed reactor (9 mm internal diameter) fitted with online GC analysis (Varian Star 3400 CX instrument with a flame ionization detector and CP-WAX 52CB 30 m × 0.32 mm × 0.5 μm capillary column), as described previously.^20^ The gas feed containing DEE vapor was obtained by passing nitrogen flow controlled by a Brooks mass flow controller through a saturator, which held liquid DEE at 0 °C (ice bath) to maintain a DEE partial pressure of 24 kPa.^32^ The DEE pressure was varied from 6–24 kPa by diluting the downstream flow with N_2_. Prior to reaction, the catalysts (typically 0.20 g, 45–180 μm particle size) were pretreated in situ at the reaction temperature for 1 h in N_2_ flow. Bulk HPW and HSiW catalysts, having high densities, were diluted with 0.1 g of silica to achieve the plug-flow regime. The downstream gas flow was analyzed by the online GC to obtain DEE conversion and product selectivity. The selectivity was defined on a carbon basis as the molar percentage of DEE converted to ethanol and ethene by taking into account reaction stoichiometry. Each catalyst test was repeated at least twice. The mean absolute percentage error in conversion and product selectivity was usually ≤5%. The reactions were carried out for 4 h time on stream (TOS) unless stated otherwise. Practically, no catalyst deactivation was observed during this time. Kinetics was studied under differential conditions (DEE conversion 2–12%). The reaction rate was calculated using the equation *R* = *XF*/*W* (mol g^–1^ h^–1^) for the differential plug-flow reactor, where *X* is the fractional conversion of DEE, *F* is the inlet molar flow rate of DEE, and *W* is the catalyst weight. The reaction was found to be of zero order in DEE with all the catalysts studied. Under such conditions, the rate does not depend on DEE conversion and is equal to the rate constant. The turnover frequency (TOF) was calculated per surface Brønsted acid site from zero-order kinetics as *R*/[H^+^], where [H^+^] is the density of surface proton sites in the catalysts. The [H^+^] values were determined as explained in the text.

## 3 Results and Discussion

### 3.1 Catalyst Characterization

The Brønsted acid catalysts used in this work included bulk and supported HPA catalysts: (i) bulk H_3_PW_12_O_40_ (HPW), H_4_SiW_12_O_40_ (HSiW), and the acidic heteropoly salt Cs_2.5_H_0.5_PW_12_O_40_ (CsPW) and (ii) silica-supported HPW and HSiW with a wide range of HPA loadings from 5.8 to 71% (Table 1). The emphasis was put on the HPA/SiO_2_ catalysts that are most interesting for practical applications.^20^ For comparison, HPW supported on TiO_2_ and ZrO_2_ at a submonolayer loading of ∼15% was also included. All these catalysts have been thoroughly characterized previously in this group using BET analysis (catalyst texture), DRIFT spectroscopy, and XRD (structural integrity) (see the Supporting Information for details, Figures S1–S7). The acid properties of catalysts (acid strength and proton site density) have been determined previously using microcalorimetry and TGA–DSC of ammonia adsorption.^20,30,33^

The surface area and porosity of bulk and supported HPA catalysts together with the texture of supports are presented in Table 1. The surface area of HPA/SiO_2_ catalysts decreases monotonically with increasing HPA loading (Figures S1 and S2). From DRIFT spectroscopy,^20,30^ the structure of Keggin units (primary structure) in all these catalysts is intact (Figures S4 and S5). Previously, it has been shown that HSiW/SiO_2_ catalysts have a higher density of surface proton sites in comparison to HPW/SiO_2_ because HSiW has more protons than HPW per Keggin unit and also a higher dispersion compared to HPW on the silica surface^20^ (Supporting Information).

**Figure 1:**
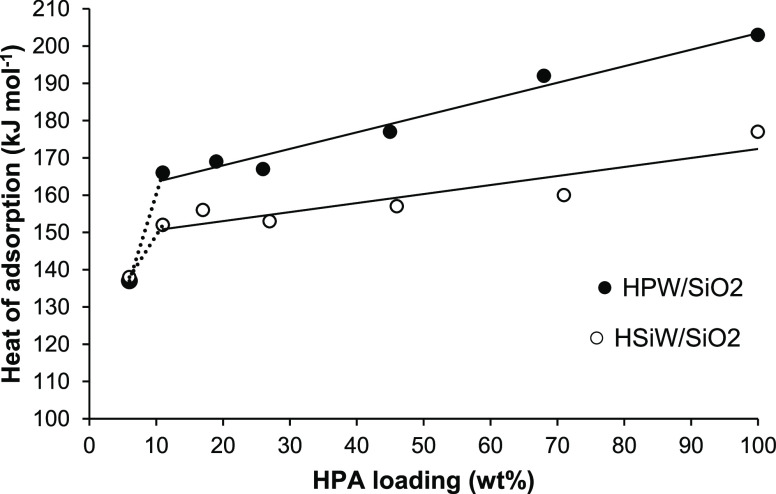
Effect of HPA loading on initial heat of NH_3_ adsorption on silica-supported HPAs.

Tungsten Keggin HPAs are well known to be the purely Brønsted acids, as demonstrated by IR spectroscopy of adsorbed pyridine, with their strength close to superacidity (refs (23−28) and references therein). The initial enthalpies of ammonia adsorption on bulk and supported HPW and HSiW catalysts that extrapolated to zero NH_3_ coverage, Δ*H*_o_, represent the strongest catalyst proton sites (Table 1). The acid strength of HPA catalysts under study decreases in the order HPW > HSiW > CsPW for bulk catalysts and HPW/SiO_2_ > HSiW/SiO_2_ > HPW/TiO_2_ > HPW/ZrO_2_ for supported catalysts.^30,33^ The acid strength decreases in the order of supports SiO_2_ > TiO_2_ > ZrO_2_, which has been explained by increasing interaction between the HPA and support in this order.^30,33^ Previously, it has been shown that the strength of HPA/SiO_2_ catalysts increases monotonically with HPA loading up to 100% loading,^20^ as illustrated in Figure 1 and presenting the Δ*H*_o_ values from Table 1. This trend has been explained by HPA–support interaction, reducing the strength of HPA proton sites at lower HPA loadings.^20^ From TPD of benzonitrile, there are no strong surface Brønsted acid sites in HSiW/SiO_2_ with low HSiW loadings of 5–10%; strong acid sites form at higher loadings, probably in the second layer of HSiW on the SiO_2_ surface.^34^

### 3.2 DEE Elimination: Thermodynamic Analysis

**Figure 2:**
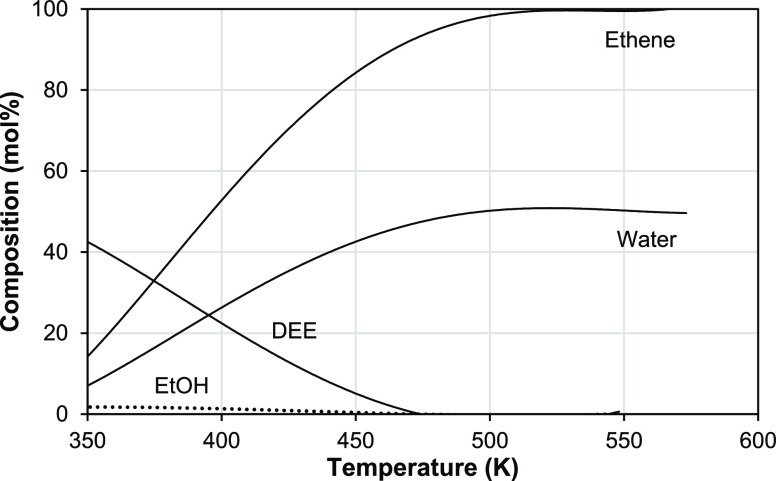
Equilibrium composition of an ideal gas system containing DEE, EtOH, C_2_H_4_, and H_2_O as a function of temperature at ambient pressure.

Figure 2 shows the composition of an ideal gas system containing DEE, EtOH, C_2_H_4_, and H_2_O at equilibrium as a function of temperature at ambient pressure calculated from thermodynamic data^32^ (see the Supporting Information for details). This diagram represents the DEE elimination system starting from pure DEE. Thermodynamic analysis shows that DEE elimination (eq 3) is less favorable than ethanol-to-ethene dehydration (eq 1), with Δ*G*^o^ = 22.3 kJ mol^–1^ (Table S2) and 7.7 kJ mol^–1^ (Table S3), respectively. The equilibrium concentration of EtOH is very small and passes a maximum at about 100 °C (Figure 2). Phung and Basca^11^ have reported thermodynamic analysis for this system starting from pure EtOH. Our analysis is in agreement with their data.

### 3.3 DEE Elimination over HPA Catalysts: DEE Conversion and Product Selectivity

Our first goal was to compare the performance of HPA catalysts in the DEE elimination with the most active catalysts reported so far such as zeolites. Since the turnover rates were not available under comparable conditions, we looked at the ethene yields per catalyst weight at comparable space velocities WHSV.

**Figure 3:**
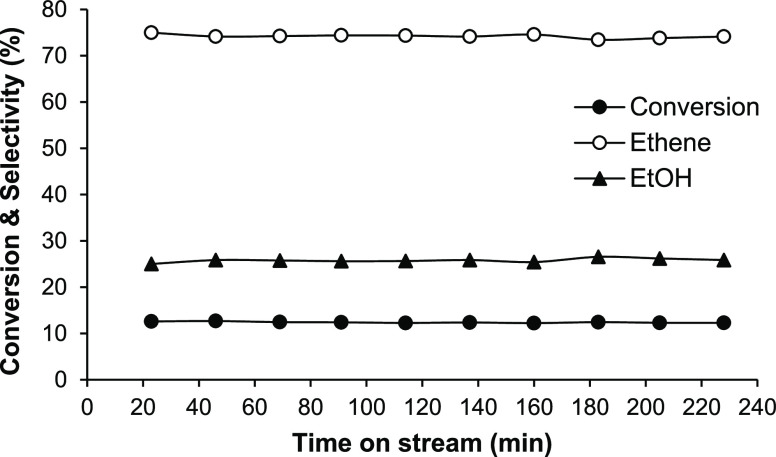
Time course for DEE elimination over 14%HSiW/SiO_2_ (0.20 g of the catalyst, 160 °C, 12 kPa DEE partial pressure, and 20 mL min^–1^ flow rate).

The HPA catalysts showed stable performance in the elimination of DEE regarding both DEE conversion and product selectivity. Practically, no catalyst deactivation was observed at 130–160 °C, as can be seen from Figure 3, showing a stable performance of the 14%HSiW/SiO_2_ catalyst at 160 °C for 4 h TOS with 12% DEE conversion. Longer-term stability tests (20 h TOS) at 200 °C showed a stable performance of 17%HPW/SiO_2_ (Figure S8) and CsPW (Figure S9) at 70–75% DEE conversion. From combustion chemical analysis of spent catalysts, less than 0.1% coke was formed on the catalysts during these reactions.

**Figure 4:**
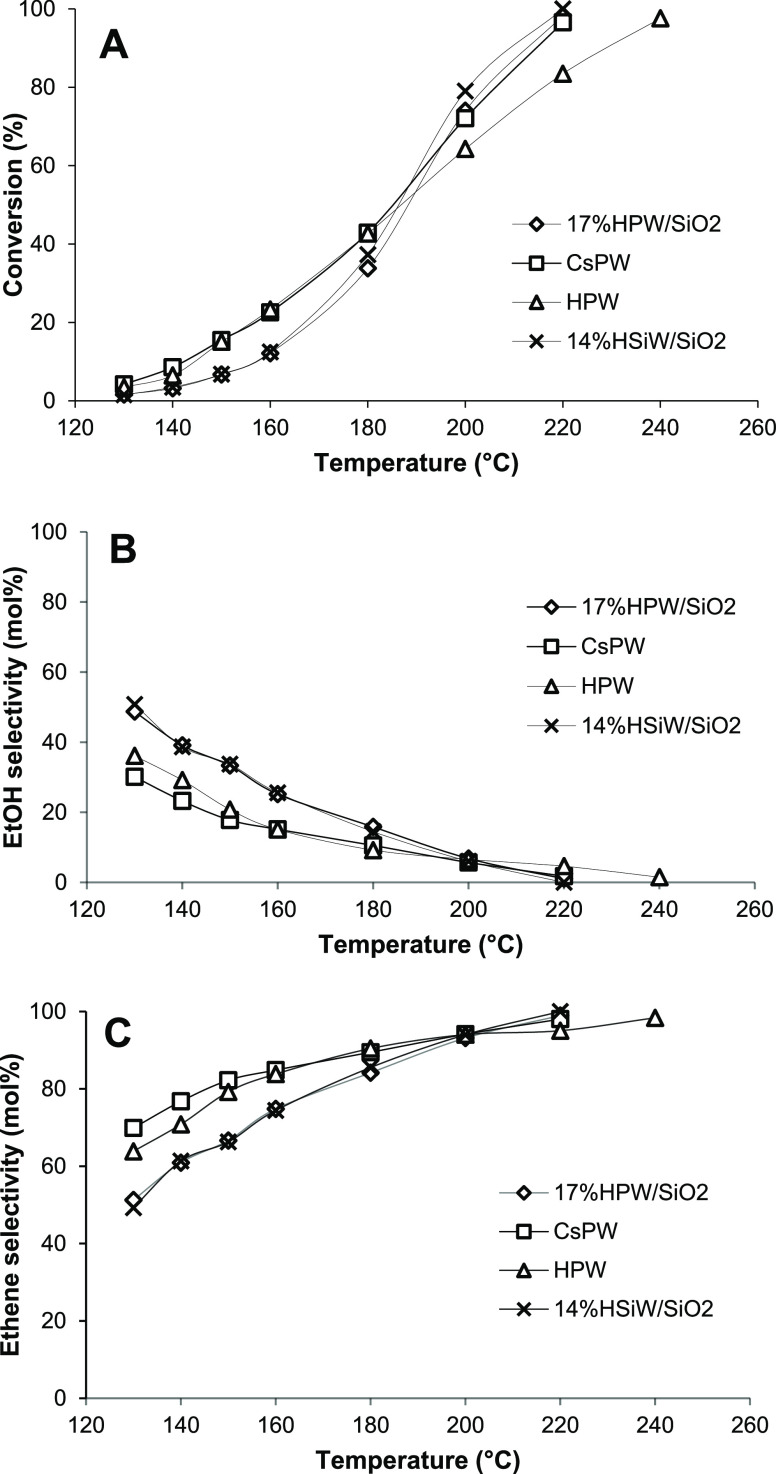
Effect of temperature on DEE conversion (A), EtOH selectivity (B), and ethene selectivity (C) (0.20 g of the catalyst, 12 kPa DEE partial pressure, 20 mL min^–1^ N_2_ flow rate, and 4 h TOS).

Figure 4 displays the effect of temperature on DEE conversion and product selectivity for bulk and silica-supported HPA catalysts. Only ethene and EtOH were observed among the organic reaction products. As expected from the thermodynamic analysis (Figure 2), the DEE conversion and ethene selectivity increase with reaction temperature, both reaching a completion at 220–250 °C, whereas EtOH selectivity decreases almost to zero. The values of DEE conversion and ethene yield at 220 °C are presented in Table 2. The most active HPA catalysts, CsPW, 17%HPW/SiO_2_, and 14%HSiW/SiO_2_, give 95, 97, and 98% ethene yield, respectively, at 97–99% DEE conversion at a space velocity WHSV = 2.2 h^–1^. These HPA catalysts outperform the best-reported zeolite catalysts.^11^ Thus, HZSM-5 (Si/Al = 140 mol/mol) and USY (Si/Al = 15 mol/mol) give 90 and 97% ethene yield, respectively, at almost 100% conversion at 350 °C and ambient pressure,^11^ that is, at a more than 100 °C higher temperature compared to the HPA catalysts. These results have been obtained at a higher WHSV = 10.4 h^–1^,^11^ but the space velocity has a relatively small effect on the ethene yield at this temperature.^11^ γ-Al_2_O_3_ and SiO_2_–Al_2_O_3_ have been found to be less efficient than HZSM-5 and USY.^11^ Previously, DEE elimination over 30%HPW/montmorillonite has been reported to give 58% ethene yield at 68% DEE conversion at 250 °C, ambient pressure, and WHSV = 1.6 h^–1^.^16^ The low activity of this catalyst can be attributed to a rather basic clay support. Basic and amphoteric supports such as MgO and Al_2_O_3_ are well known to decrease the acidity of HPAs and may even cause disintegration of the HPA structure.^24−26^ Silica is most frequently used for supporting HPAs because of its inertness toward HPA and availability in a wide textural variety.^20,24−26^

**Table 2:** DEE Conversions and Ethene Yields^a^

	14%HSiW/SiO_2_	17%HPW/SiO_2_	CsPW	HPW	HSiW
DEE conv. (%)	99	98	97	83	72
C_2_H_4_ yield (%)	98	97	95	79	67

a 220 °C, 12 kPa DEE partial pressure, 20 mL min^–1^ flow rate, 0.20 g of the catalyst, and WHSV = 2.2 h^–1^ (for HPW and HSiW, 0.30 g of the catalyst and WHSV = 1.5 h^–1^). Proton site densities for these catalysts are given in Table 4.

### 3.4 Kinetics of DEE Elimination

Kinetics of DEE elimination was studied under differential conditions (DEE conversion 2–12%), 130–160 °C and 6–24 kPa DEE partial pressure. The reactions were carried out for 4 h TOS, during which no catalyst deactivation was observed (Figure 3). Comparison of the data in Figure 4 with the thermodynamic data in Figure 2 shows that the reaction system was far from equilibrium at 130–160 °C. Thus, at 150 °C (423 K), the equilibrium conversion of DEE is ∼90% (Figure 2), whereas in our reaction system, it was 7–15% (Figure 4A).

**Figure 5:**
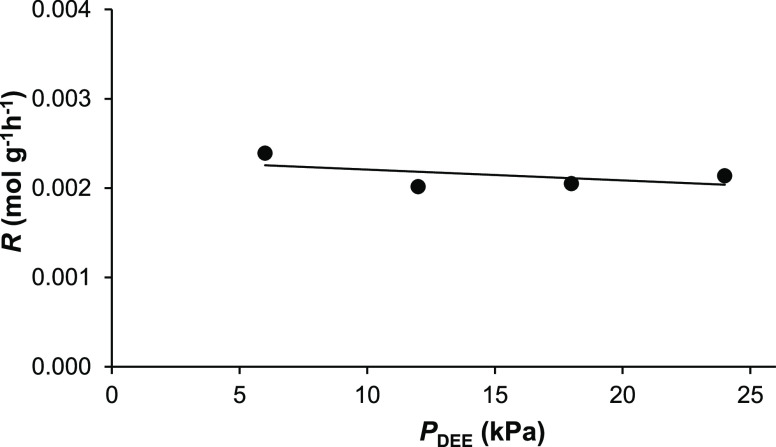
Effect of DEE partial pressure on the rate of DEE elimination over 14%HSiW/SiO_2_ (0.20 g of the catalyst, 150 °C, 20 mL min^–1^ flow rate, and 4 h TOS).

For all HPA catalysts, the reaction was found to be close to zero order in DEE, as illustrated in Figure 5 for 14%HSiW/SiO_2_, where the rate of DEE conversion remains almost constant (0.0020–0.0024 mol g^–1^ h^–1^) at a fivefold variation of DEE partial pressure (5–25 kPa). Close to zero-order logarithmic plots for all HPA catalysts are shown in Figure S10, with the order in DEE varying from −0.05 to 0.14. Assuming Langmuir-type kinetics, this would indicate that the active sites in HPA catalysts were saturated with adsorbed DEE molecules.

**Figure 6:**
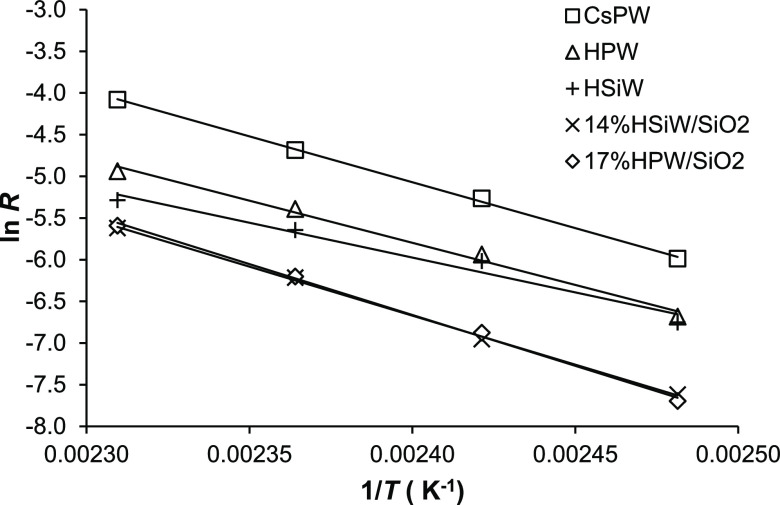
Arrhenius plots for DEE elimination over HPA catalysts (0.055–0.20 g catalyst amount, 12 kPa DEE partial pressure, 20 mL min^–1^ flow rate, WHSV = 2.2–8.0 h^–1^, and 130–160 °C temperature range; *R* is the reaction rate per total catalyst weight in mol g^–1^ h^–1^).

Figure 6 shows the Arrhenius plots for bulk and silica-supported HPA catalysts. The activation energies (*E*) obtained were in the range of 69.5 to 101.5 kJ mol^–1^ (Table 3). Given the zero reaction order in DEE, the observed activation energies are the true value *E*. For HPW supported on montmorillonite, *E* = 80.6 kJ mol^–1^ has been reported.^16^ The high *E* values and zero order in DEE indicate that the reactions were not affected by diffusion limitations. The absence of internal diffusion limitations is also supported by the Weisz–Prater analysis.^35^ For example, for 17%HPW/SiO_2_ at 150 °C, the Weisz–Prater criterion was calculated to be *C*_WP_ = 1.1 × 10^–4^ ≪ 1, indicating no internal diffusion limitations (see the Supporting Information for details).

**Table 3:** Activation Energies (in kJ mol^–1^) for HPA Catalysts^a^

17%HPW/SiO_2_	14%HSiW/SiO_2_	CsPW	HPW	HSiW
101.5	97.8	91.5	84.1	69.5

a At 130–160 °C, 12 kPa DEE partial pressure, 20 mL min^–1^ flow rate, and 0.055–0.20 g of the catalyst (WHSV = 2.2–8.0 h^–1^).

Assuming that in the overall DEE-to-ethene conversion, the elimination of DEE to ethene and EtOH (eq 3, Scheme 1) is the irreversible rate-limiting step with equilibrated readsorption of EtOH, followed by irreversible EtOH dehydration (eq 1), with no readsorption of products taking place, and the observed rate of DEE conversion is given by the Langmuir-type eq 4(12)
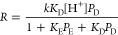
4

Here, *K*_E_ and *K*_D_ are the equilibrium constants for EtOH and DEE adsorption on Brønsted acid sites, respectively, *k* is the rate constant of the rate-limiting step, [H^+^] is the density of accessible proton sites in the catalyst, and *P*_E_ and *P*_D_ are the partial pressures of EtOH and DEE, respectively. At high *P*_D_, when catalyst active sites are saturated with the ether, the reaction becomes zero order in DEE, that is, *R* = *k*[H^+^], in agreement with the experimental data. From this data, the true values of TOF can be obtained as *R*/[H^+^] (see below).

### 3.5 Mechanistic Considerations

Misono et al.^23^ proposed two types of mechanisms for heterogeneous acid catalysis by HPA—surface-type and bulk-type mechanisms. The surface type is the conventional acid catalysis involving proton sites on the surface of HPA. The bulk type (bulk type I)^23^ is suggested to operate in the case of bulk HPAs with polar reactants, such as lower alcohols, ethers, ketones, and so forth, which are capable of being absorbed in large quantities into catalyst bulk in the interstitial space between heteropoly anions. In this case, all HPA protons, both bulk and surface ones, are thought to be accessible so that reaction occurs pseudo-homogeneously, and its rate should scale with the total number of HPA protons or HPA weight. Bulk-type catalysis has been demonstrated for MeOH dehydration over bulk HPW in a static system.^25,36,37^ However, as Moffat argued (ref (25) p 71), the bulk-type reaction via substrate absorption into the interstitial space would be almost inevitably diffusion-hindered in a steady-state flow system. In contrast, nonpolar reactants, for example, hydrocarbons, that cannot penetrate HPA bulk, react via the surface-type mechanism.^23^

DEE is a relatively polar solvent, which readily dissolves HPAs such as HPW and HSiW and is capable of absorbing into the bulk of HPA crystallites. Hypothetically, DEE might react with bulk HPA and high-loaded HPA/SiO_2_ catalysts via the bulk-type mechanism. Therefore, here, we attempted to gain an insight into the mechanism of DEE elimination over HPA/SiO_2_ catalysts regarding the possibility of bulk-type versus surface-type catalysis.

With silica-supported HPAs at low and medium HPA loadings, both polar and nonpolar substrates typically react via the surface-type catalysis, showing similar dependencies between catalyst activity and HPA loading—the activity, expressed as substrate conversion or reaction rate per total weight of HPA/SiO_2_ catalyst, increases with HPA loading at a constant HPA/SiO_2_ catalyst weight, reaching a plateau at 25–40% loading, or passes a maximum.^26,34,38^ This is the result of a trade-off between the density of surface proton sites and their strength at varying HPA loading. The proton site density (per total catalyst weight) passes a maximum at a medium HPA loading due to the sharp decrease in catalyst surface area to less than 10 m^2^ g^–1^ for bulk HPA.^20,34^ At the same time, the strength of proton sites increases monotonically with HPA loading (Figure 1). Using the TPD of benzonitrile, which interacts only with the surface acid sites in HPA, the amount of strong surface Brønsted sites in HSiW/SiO_2_ has been found to pass a maximum at a HSiW loading of ∼50%.^34^

**Figure 7:**
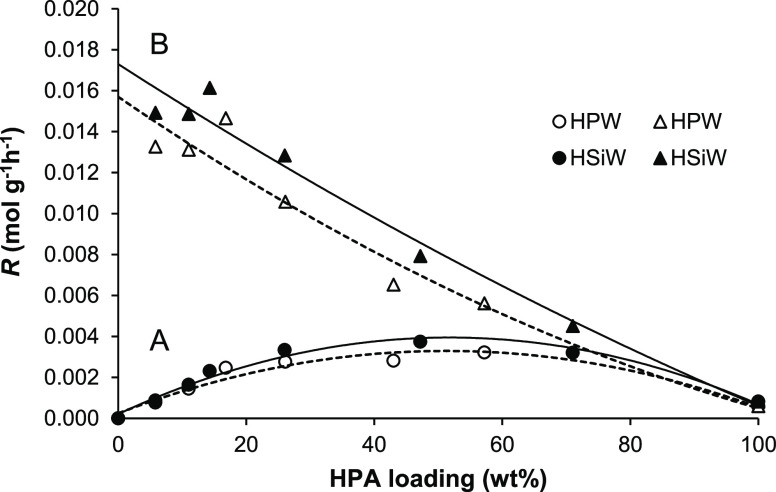
Plot of MeOH dehydration rate per total HPA/SiO_2_ catalyst weight (A, open and closed circles) and per HPA weight (B, open and closed triangles) versus HPA loading (120 °C and WHSV = 0.30 h^–1^).

For the surface-type mechanism, in the case of reactions less demanding toward catalyst acid strength, the activity of supported HPA is expected to scale with the proton surface site density passing a maximum as the HPA loading increases. Such a dependence has been observed for the dehydration of MeOH and EtOH over HPA/SiO_2_ at 120 °C, which suggests the surface-type mechanism operating in the whole range of HPA loading including high-loaded and bulk HPA catalysts.^20^Figure 7, compiled from the data,^20^ shows that the rate of MeOH dehydration per HPA/SiO_2_ catalyst weight passes a maximum as the HPA loading increases from 0 to 100% at a constant catalyst weight, whereas the rate per HPA weight decreases in parallel with decreasing number of surface proton sites, as expected for the surface-type catalysis. The surface-type mechanism for MeOH and EtOH dehydration is also supported by Brønsted relation between the catalyst activity and acid strength, which holds for both bulk HPAs and other catalysts operating via the surface-type mechanism (oxide-supported HPAs with submonolayer HPA loadings, CsPW, and zeolites).^20,33^ On the other hand, for more demanding reactions, for example, the isomerization of cycloalkanes over HPW/SiO_2_ occurring at 300 °C via the surface-type mechanism, the activity tends to plateau at higher HPW loadings due to the competing effect of increasing proton site strength.^25,39^ Contrary to the surface type catalysis, for the bulk-type one, the rate per total catalyst weight is expected to increase with HPA loading, whereas the rate per HPA weight should remain approximately constant at varying HPA loading.

**Figure 8:**
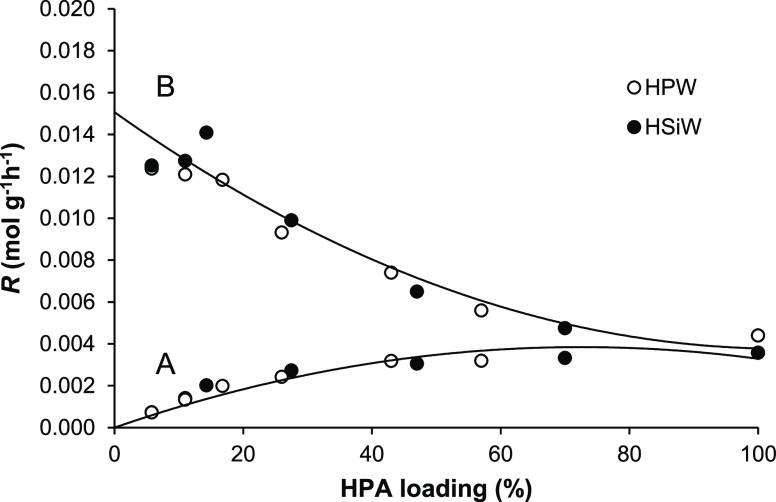
Plot of DEE elimination rate per total HPA/SiO_2_ catalyst weight (A) and per HPA weight (B) versus HPA loading (0.20 g of the catalyst, 150 °C, 12 kPa DEE partial pressure, and 20 mL min^–1^ flow rate).

Figure 8 shows the rate of DEE elimination per total HPA/SiO_2_ catalyst weight as a function of HPA loading for HPW/SiO_2_ and HSiW/SiO_2_ catalysts at 150 °C. The rate increases with HPA loading up to about 25% loading and levels off at higher loadings. As seen, this plot is somewhat different from that for MeOH dehydration (Figure 7). In fact, it is as expected for the surface-type mechanism for the more demanding DEE elimination occurring at 150 °C. The rate of DEE elimination per HPA weight decreases with increasing HPA loading (Figure 8B), as for MeOH dehydration. Therefore, these results are consistent with DEE elimination occurring via the surface-type mechanism in the whole range of HPA loading including high-loaded and bulk HPA catalysts. This cannot completely rule out the participation of bulk protons located near the surface of HPA, but these do not seem to play a significant role. This conclusion is also supported by Brønsted relation between the catalyst activity and acid strength (see below).

It is worth noting that HPW and HSiW catalysts exhibit very close activity per catalyst weight over the whole range of HPA loading (Figure 8) despite HPW catalysts having stronger acid sites than HSiW ones (Table 1). This may be explained by the higher proton surface site density in HSiW catalysts due to the larger number of protons per Keggin unit and the higher HSiW dispersion on the silica surface compared to HPW catalysts.^20^ Nevertheless, the turnover activity (TOF) of HPW catalysts was found to be greater than that of HSiW ones, as expected from their acid strength (see below).

**Figure 9:**
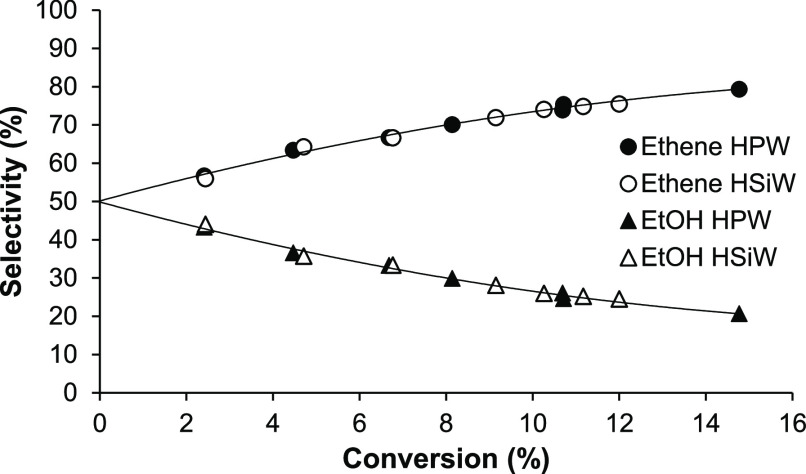
Plot of product selectivity versus DEE conversion for HPA/SiO_2_ catalysts with HPA loading varied from 5.8 to 100% (0.20 g of the catalyst, 150 °C, 12 kPa DEE partial pressure, and 20 mL min^–1^ flow rate).

Figure 9 presents the data shown in Figure 8 from a different angle—as product selectivity versus DEE conversion. Both HPW/SiO_2_ and HSiW/SiO_2_ catalysts give practically the same ethene and EtOH selectivities. As the DEE conversion increases with increasing HPA loading, the selectivity to ethene grows at the expense of EtOH. Importantly, the extrapolation to zero conversion gives a 50:50 ethene/EtOH selectivity, which shows that the initial reaction step is DEE → C_2_H_4_ + EtOH. This demonstrates that the DEE-to-ethene conversion occurs through the consecutive pathway: DEE → C_2_H_4_ + EtOH followed by EtOH → C_2_H_4_ + H_2_O, where ethene is both a primary product of DEE cracking and a secondary product via dehydration of the primary product EtOH. The mechanism of ethene formation from EtOH dehydration is still debated (ref (10) and references therein). DEE forms in EtOH dehydration at low temperatures, whereas ethene forms at higher temperatures. The question is whether the ethene forms directly from EtOH or via DEE cracking or both.^10^ Our results, therefore, show that ethene can form via the DEE cracking.

### 3.6 Relationship between the Turnover Rate and Catalyst Acid Strength

Table 4 shows the density of catalyst surface proton sites and TOF values for the elimination of DEE at 150 °C for all the HPA catalysts studied including bulk catalysts (HPW, HSiW, and CsPW) and supported catalysts at a submonolayer HPA loading of 14–17%. The TOF values were calculated per surface proton site from zero-order kinetics as *R*/[H^+^]. The densities of surface proton sites were estimated as described elsewhere.^20,30,32^ For supported HPA catalysts with submonolayer HPA coverage, all stoichiometric HPA protons were assumed to be equally available for reaction. This is supported by ammonia adsorption calorimetry^20,40^ and titration with di-*tert*-butylpyridine.^19,20^ For bulk catalysts, HPW, HSiW, and CsPW, the number of surface protons was estimated using a Keggin unit cross section of 144 Å^2^ and the catalyst surface areas from Table 1 with the stoichiometric number of protons per Keggin unit.^23−26^ The TOF values thus obtained should be regarded as an approximation since the number of accessible protons could differ from the stoichiometric numbers used in the calculations. The TOF values thus calculated range from 4.3 h^–1^ for 14%HPW/ZrO_2_ to 170 h^–1^ for bulk HPW, indicating a strong effect of catalyst acid strength on the turnover reaction rate as can be seen from the Δ*H*_o_ values for these catalysts.

**Table 4:** Proton Site Density and TOF for HPA Catalysts

catalyst	[H^+^] (mmol g^–1^)^a^	TOF (h^–1^)^b^	–Δ*H*_o_ (kJ mol^–1^)^c^
HPW	0.0198	170	203
HSiW	0.0369	65	177
CsPW	0.0750	53	164
17%HPW/SiO_2_	0.175	13	167
14%HSiW/SiO_2_	0.195	11	152
15%HPW/TiO_2_	0.152	6.9	143
14%HPW/ZrO_2_	0.146	4.3	121

a Density of surface proton sites per total catalyst weight.

b TOF per surface proton site at 150 °C, 24 kPa DEE partial pressure, and 20 mL min^–1^ flow rate.

c Initial enthalpy of NH_3_ adsorption from ammonia adsorption microcalorimetry at 150 °C (±6 kJ mol^–1^).^20,30^

**Figure 10:**
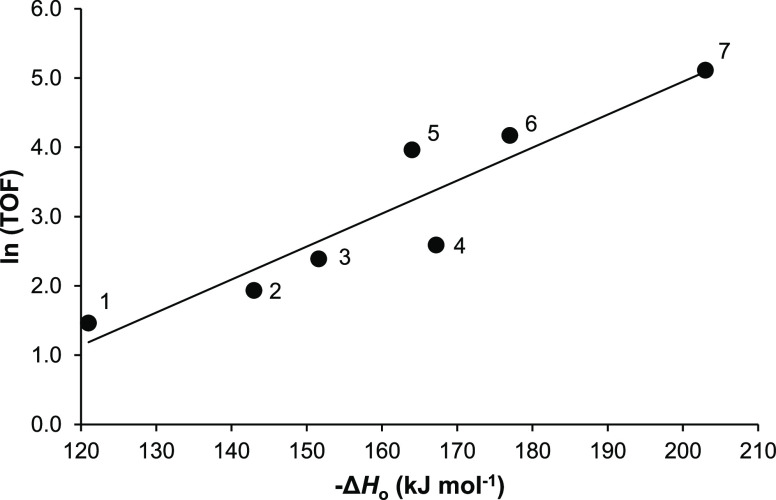
Plot of ln(TOF) for DEE elimination (TOF in h^–1^) over HPA catalysts versus initial heat of NH_3_ adsorption (150 °C, 0.20 g of the catalyst, 24 kPa DEE partial pressure, and 20 mL min^–1^ flow rate): (1) 14%HPW/ZrO_2_, (2) 15%HPW/TiO_2_, (3) 14%HSiW/SiO_2_, (4) 17%HPW/SiO_2_, (5) CsPW, (6) HSiW, and (7) HPW.

Figure 10 shows a Brønsted linear relation between the turnover activity of HPA catalysts in DEE elimination, ln (TOF), and their acid strength determined from the initial enthalpy of NH_3_ adsorption, Δ*H*_o_. Both parameters were determined at the same temperature of 150 °C and similar flow conditions. As seen, the relation holds for the bulk HPAs and for the oxide-supported HPAs—all being the Brønsted acid catalysts. This implies that Brønsted acid sites play an important role in the elimination of DEE over HPA catalysts. It also implies the same reaction mechanism for the whole series of catalysts involved.^41^ Since this relation holds for the oxide-supported HPAs with submonolayer HPA loadings and CsPW operating via the surface-type mechanism, on the one hand, and for the bulk HPAs, on the other, it suggests that the bulk HPW and HSiW would also largely operate through the mechanism of surface catalysis, in agreement with our conclusion in Section 3.5.

## 4 Conclusions

DEE is the key intermediate of ethanol-to-ethene dehydration. In the presence of acid catalysts, DEE undergoes elimination to produce ethene and EtOH; the latter dehydrates to ethene. Knowledge about the elimination of DEE can shed light on the mechanism of the ethanol-to-ethene dehydration and help to optimize the production of ethene.

In this work, the elimination of DEE has been studied at a gas–solid interface over a range of bulk and supported Brønsted solid acid catalysts based on tungsten Keggin HPAs in a fixed-bed reactor at 130–250 °C and ambient pressure. The most active HPA catalysts are silica-supported H_3_PW_12_O_40_ and H_4_SiW_12_O_40_ and the bulk heteropoly salt Cs_2_._5_H_0_._5_PW_12_O_40_, giving 95–98% ethene yield at 220 °C and WHSV = 2.2 h^–1^. The HPA catalysts outperform the best-reported zeolite catalysts such as HZSM-5 and USY, which give the same yield but at temperatures about 100 °C higher compared to the HPA catalysts. A Brønsted correlation between the turnover catalyst activity and catalyst acid strength has been established, which indicates that Brønsted acid sites play an important role in the elimination of DEE over HPA catalysts. The results obtained point to the DEE conversion occurring through the consecutive reaction pathway: DEE → C_2_H_4_ + EtOH followed by EtOH → C_2_H_4_ + H_2_O, where ethene is both a primary product of DEE elimination and a secondary product via dehydration of the primary product EtOH. Evidence is provided that DEE elimination over bulk HPA and high-loaded HPA/SiO_2_ catalysts proceeds via a surface-type mechanism.

## Supporting Information Available

Catalyst characterization (BET, DRIFTS, and XRD data); thermodynamic calculations; reaction time courses; kinetic data (reaction order in DEE); and Weisz–Prater analysis (PDF)
