# Supplementary material for: Diethyl Ether Conversion to Ethene and Ethanol Catalyzed by Heteropoly Acids
Source: ACS Omega. 2021 Mar 23;6(13):9310–8. doi: 10.1021/acsomega.1c00958 (PMC8028136; doi:10.1021/acsomega.1c00958)
Supplement: Supplementary file 1 — ao1c00958_si_001.pdf [file ao1c00958_si_001.pdf]

Diethyl ether conversion to ethene and ethanol catalysed by heteropoly acids

Rawan Al-Faze,[a,b] Elena F. Kozhevnikova,[a] Ivan V. Kozhevnikov[a,*]

* Corresponding author. Tel.: +44(0)1517942938
E-mail address: kozhev@liverpool.ac.uk (I. V. Kozhevnikov)

[a]*University of Liverpool, Department of Chemistry, Liverpool L69 7ZD, UK*

[b]*Taibah University, Department of Chemistry, P.O Box 30002, Al-Madinah Al-Munawarah 41147, Saudi Arabia*

Supporting information

Catalyst texture and HPA structural integrity[1]

The surface area and porosity of bulk HPAs and supported HPA catalysts prepared by wet impregnation of support with an aqueous HPA solution is presented in Table 1, together with the texture of supports. The catalyst surface area (per gram of catalyst) decreases monotonously with increasing HPA loading. Analysis shows that this is mainly the result of the addition of dense HPAs to the silica without significant change of the pore structure. Figures S1–S2 show that HPA loading had only a small effect on the surface area and the pore volume per gram of silica up to ~70% HPA loading, which corresponds to an average HPA surface coverage of ~2 monolayers (calculated assuming an HPA cross section of 144 $Å^2$ and the surface area of Aerosil 300 silica support of ~300 m^2/g). The pore diameter practically did not change up to ~50% HPA loading (Figure S3) This suggests that HPAs did not block the pores in silica support, at least in the range of 0–50% HPA loading which is the most important for practical use of these catalysts.[1]

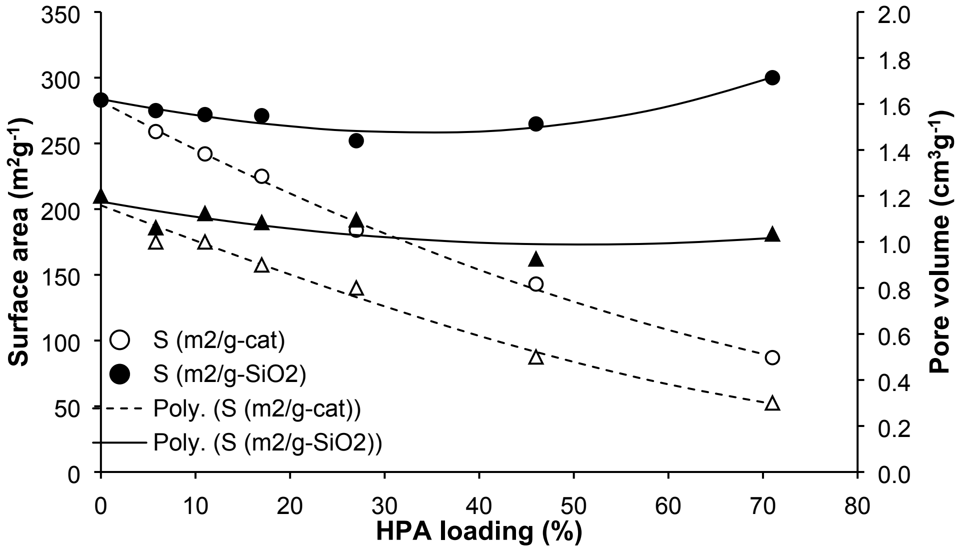

Figure S1. Effect of HSiW loading on surface area and pore volume of HSiW/SiO_2 catalysts (open markers represent the values per gram of catalyst, solid markers per gram of SiO_2).

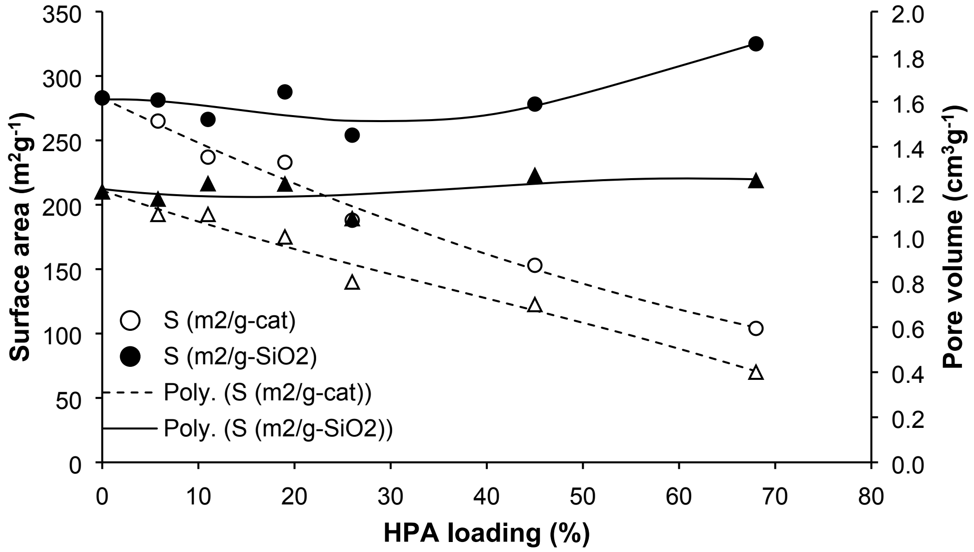

Figure S2. Effect of HPW loading on surface area and pore volume of HPW/SiO_2 catalysts (open markers represent the values per gram of catalyst, solid markers per gram of SiO_2).

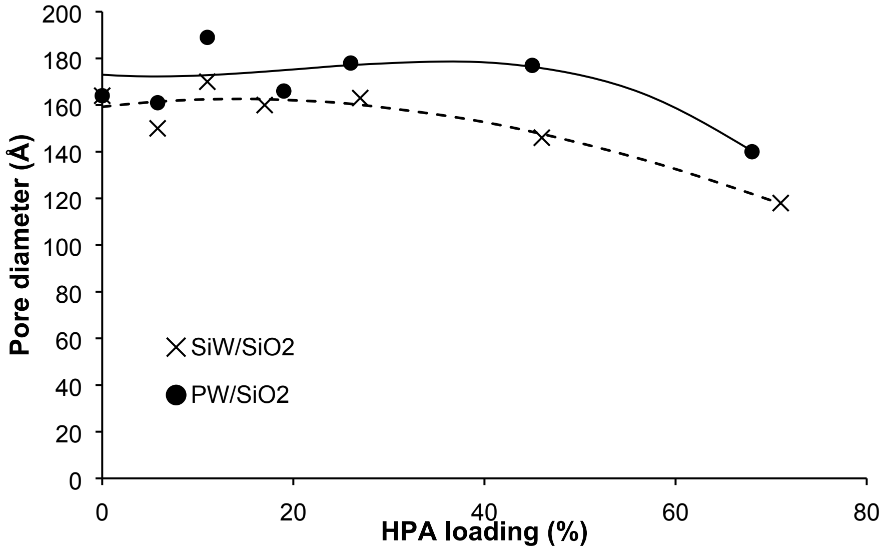

Figure S3. Effect of HPA loading on pore diameter of HPA/SiO_2 catalysts.

DRIFT spectra for HPW/SiO_2 and HSiW/SiO_2 catalysts together with the spectra of bulk HPW and HSiW are shown in Figures S4–S5. Bulk HPAs display the well-known infrared bands of metal-oxygen stretching vibrations in the range of 700–1100 cm^{-1} characteristic of Keggin heteropoly anions. HPW shows four bands at 808 cm^{-1} (W–O–W edge-sharing), 889 cm^{-1} (W–O–W corner-sharing), 984 cm^{-1} (W=O) and 1082 cm^{-1} (P–O). The corresponding bands for bulk HSiW occur at 792 cm^{-1} (W–O–W edge-sharing), 881 cm^{-1} (W–O–W corner-sharing), 927 cm^{-1} (Si–O) and 980 cm^{-1} (W=O). It can be seen that these bands are present unchanged in the spectra of silica-supported HPAs, except for the bands of P–O at 1082 cm^{-1} and W–O–W at 808 cm^{-1} for HPW/SiO_2 and the band of W–O–W at 792 cm^{-1} for HSiW/SiO_2, which are obscured by the intense bands of silica centred at 804 and 1108 cm^{-1}. This shows that the structure of Keggin units (primary structure) in all HPA catalysts is largely intact, in agreement with previous report.[1]

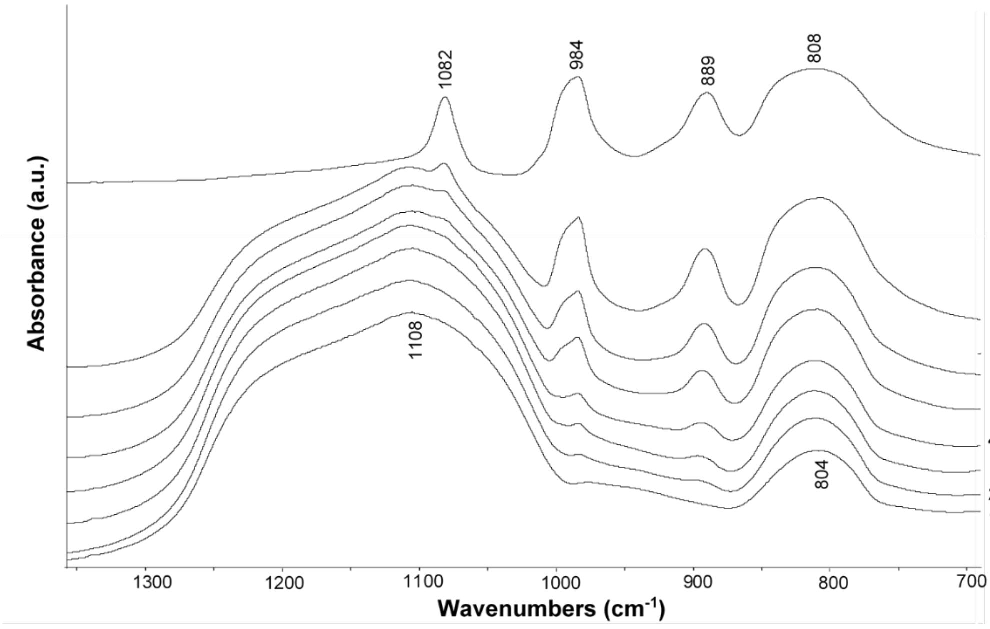

Figure S4. DRIFT spectra of HPW/SiO_2 catalysts (KBr mixtures): (1) SiO_2, (2) 5.8%HPW/SiO_2, (3) 11%HPW/SiO_2, (4) 19%HPW/SiO_2, (5) 26%HPW/SiO_2, (6) 45%HPW/SiO_2, (7) 68%HPW/SiO_2 and (8) bulk HPW.

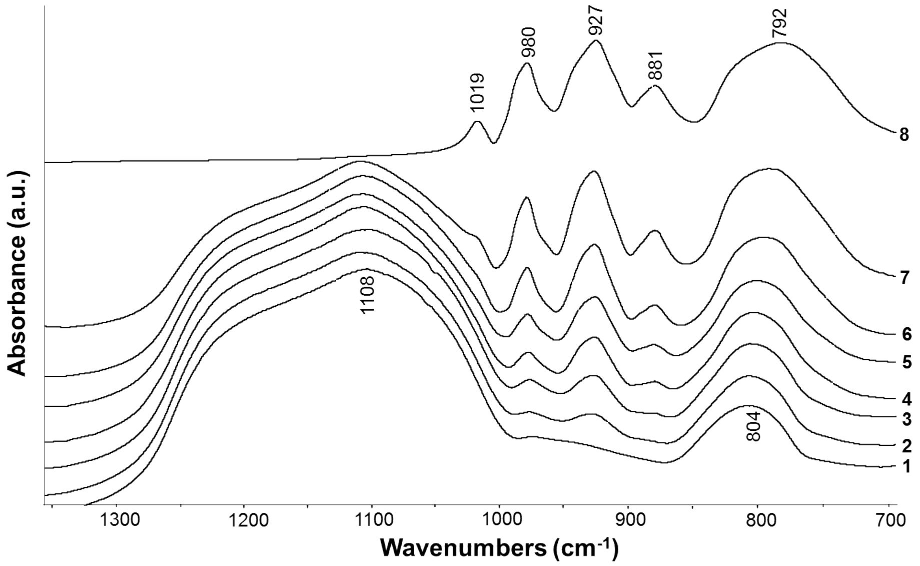

Figure S5. DRIFT spectra of HSiW/SiO_2 catalysts (KBr mixtures): (1) SiO_2, (2) 5.8%HSiW/SiO_2, (3) 11%HSiW/SiO_2, (4) 17%HSiW/SiO_2, (5) 27%HSiW/SiO_2, (6) 46%HSiW/SiO_2, (7) 71%HSiW/SiO_2 and (8) bulk HSiW.

XRD patterns for HPW/SiO_2 and $HSiW/SiO_2$ are shown in Figures S6–S7. In HPW/SiO_2 catalysts, HPW crystal phase appears from 11% HPW loading, whereas in $HSiW/SiO_2$ catalysts, HSiW crystallites are seen from a higher HSiW loading of 17%. The HPA crystal phase in supported catalysts exhibits the diffraction patterns exactly matching those for the bulk HPAs. This confirms the structural integrity of HPW and HSiW on the silica surface, i.e. the same crystal structure of bulk and supported HPA crystallites. The average size of HPA crystallites on the silica surface was estimated from the Scherrer equation.[1] As expected, the size of HPW and HSiW crystallites increased with HPA loading. Notably, the HSiW crystallites were smaller than the HPW ones at similar HPA loadings, indicating a higher HSiW dispersion on the silica surface in comparison to HPW. These results point to a higher density of surface proton sites in $HSiW/SiO_2$ catalysts compared to HPW/SiO_2 at equal HPA loadings. This is not only because HSiW has more protons than HPW per Keggin unit, but also due to the higher dispersion of HSiW on the silica surface compared to HPW.[1]

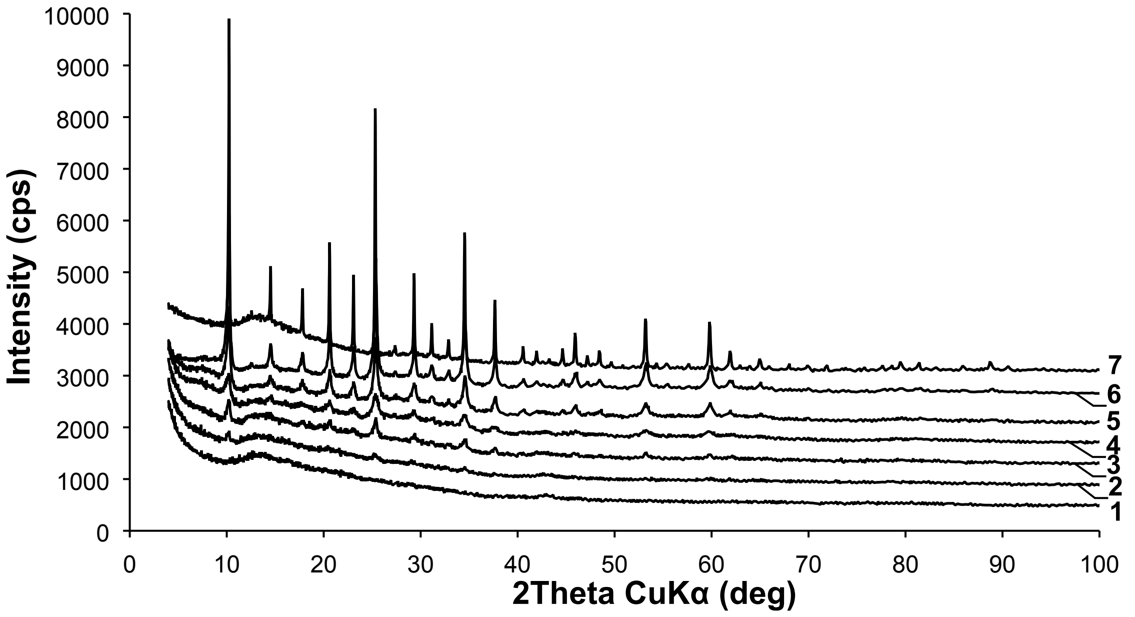

Figure S6. XRD patterns for HPW/SiO_2 with different HPW loadings: (1) 5.8%, (2) 11%, (3) 19%, (4) 26%, (5) 45%, (6) 68% and (7) bulk HPW.

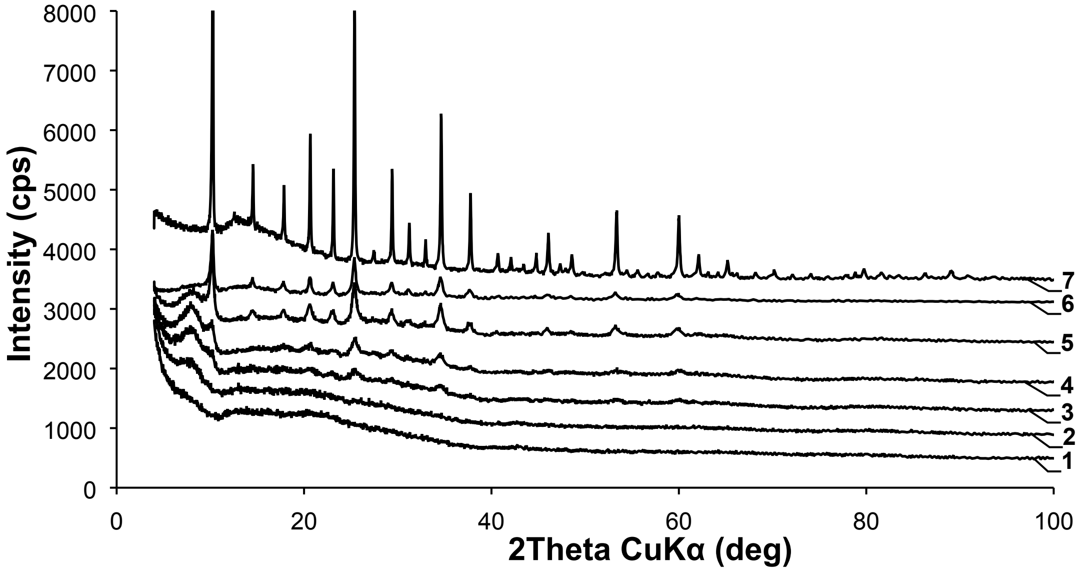

Figure S7. XRD patterns for HSiW/SiO_2 with different HSiW loadings (1) 5.8%, (2) 11%, (3) 17%, (4) 27%, (5) 46%, (6) 71% and (7) bulk HSiW.

Thermodynamic calculations

The thermodynamic analysis includes calculation of the Gibbs free energy, equilibrium constant and equilibrium conversion for diethyl ether (DEE) elimination (eq. (1) and (2)) and ethanol-to-ethene dehydration (eq. (3)) in the ideal gas system at 1 bar pressure. Initial thermodynamic data on the formation functions $\Delta_f G^o$ and $\Delta_f H^o$ together with S^o and C_p at standard conditions (298.15 K and 1 bar) are presented in Table S1.

$$DEE = EtOH + C_2H_4 \qquad (1)$$

$$DEE = 2C_2H_4 + H_2O \qquad (2)$$

$$EtOH = C_2H_4 + H_2O \qquad (3)$$

Table S1. Initial thermodynamic data (298.15 K, 1 bar, ideal gas phase).[a]

Compound	$\Delta_f G^o$ kJ/mol	$\Delta_f H^o$ kJ/mol	S^o J/mol K	C_p J/mol K
H_2O	–228.6	–241.8	188.8	33.6
C_2H_4	68.4	52.4	219.3	42.9
EtOH	–167.9	–234.8	281.6	65.6
Et_2O		–252.1	342.7	119.5

[a] CRC Handbook of Chemistry and Physics, 84th ed., 2003–2004.

Equations used for the calculations are given below, where K_p is the equilibrium constant and x is the equilibrium conversion. ΔC_p was assumed to be independent of temperature, i.e., $\Delta C_p = \Delta C_p^o$. The results are presented in Tables S2, S3 and S4.

$$\Delta H = \Delta H^o + \Delta C_p^o(T - 298.15)$$

$$\Delta S = \Delta S^o + \Delta C_p^o \ln(T/298.15)$$

$$\Delta G = \Delta H - T\Delta S$$

$$K_p = \exp\{-\Delta G/RT\}$$

For equations (1) and (3):

$K_p = x^2P/(1-x^2)$, where P is the total pressure (1 bar).

$$x = \sqrt{K_p/(P + K_p)}$$

For equation (2):

$$K_p = \frac{4x^3P^2}{(1-x)(1+2x)^2}$$

From this equation x was calculated by the trial and error method.

Table S2. Thermodynamics of DEE elimination (DEE = EtOH + C_2H_4).[a]

T °C	T K	ΔH kJ/mol	ΔS J/mol K	ΔG kJ/mol	K_p bar	x
25	298.15	69.7	158.2	22.53	1.13E-04	0.011
75	348.15	69.2	156.5	14.67	6.30E-03	0.079
125	398.15	68.6	155.0	6.88	1.25E-01	0.334
175	448.15	68.1	153.7	–0.84	1.25E+00	0.746
225	498.15	67.5	152.6	–8.49	7.78E+00	0.941
275	548.15	67.0	151.5	–16.10	3.42E+01	0.986
300	573.15	66.7	151.0	–19.88	6.48E+01	0.992

[a]At 1 bar, ΔC_p^o = –11.0 J/mol K.

Table S3. Thermodynamics of EtOH-to-ethene dehydration (EtOH = C_2H_4 + H_2O).[a]

T °C	T K	ΔH kJ/mol	ΔS J/mol K	ΔG kJ/mol	K_p bar	x
25	298.15	45.4	126.5	7.68	4.51E-02	0.208
75	348.15	46.0	128.2	1.37	6.24E-01	0.620
125	398.15	46.5	129.7	–5.14	4.72E+00	0.908
175	448.15	47.0	130.9	–11.66	2.29E+01	0.979
225	498.15	47.6	132.1	–18.21	8.11E+01	0.994
275	548.15	48.1	133.1	–24.86	2.34E+02	0.998
300	573.15	48.4	133.6	–28.17	3.70E+02	0.999

[a]At 1 bar, ΔC_p^o = 10.9 J/mol K.

The DEE dehydration (eq. (2)) is the sum of reactions (1) and (3). Thermodynamic data for reaction (2) were obtained by combining the data from Tables S2 and S3 and shown in Table S4.

Table S4. Thermodynamics of DEE dehydration (DEE = $2C_2H_4$ + H_2O).

T °C	T K	ΔH kJ/mol	ΔS J/mol K	ΔG kJ/mol	K_p bar^2	x
25	298.15	115.1	284.7	30.22	5.08E–06	0.016
75	348.15	115.1	284.7	15.98	4.00E–03	0.10
125	398.15	115.1	284.7	1.748	5.90E–01	0.52
175	448.15	115.1	284.7	–12.49	2.85E+01	0.96
225	498.15	115.1	284.6	–26.72	6.33E+02	0.998
275	548.15	115.1	284.6	–40.95	7.99E+03	1.000

300	573.15	115.1	284.6	–48.07	2.40E+04	1.000

From the data in Tables S2 and S4, the equilibrium composition of ideal gas system containing DEE, EtOH, C_2H_4 and H_2O was calculated. The results are given in Table S5.

Table S5. Equilibrium composition of ideal gas system containing DEE, EtOH, C_2H_4 and H_2O (mol% based on C_2H_4) as a function of temperature at ambient pressure starting from pure DEE.

T	*T*	Equilibrium composition				Comment
°C	K	DEE	EtOH	C_2H_4	H_2O	
0	273.15	50.0	0	0	0	
25	298.15	49.0	0.5	0.5	0	from eq. (1)
75	348.15	46.0	1.5	2.5	2.5	from eq. (1), (3)
125	398.15	24.0	0	52.0	26.0	from eq. (2)
175	448.15	2.0	0	96.0	48.0	from eq. (2)
225	498.15	0.1	0	99.8	49.9	from eq. (2)
275	548.15	0	0	100	50	from eq. (2)
300	573.15	0	0	100	50	from eq. (2)

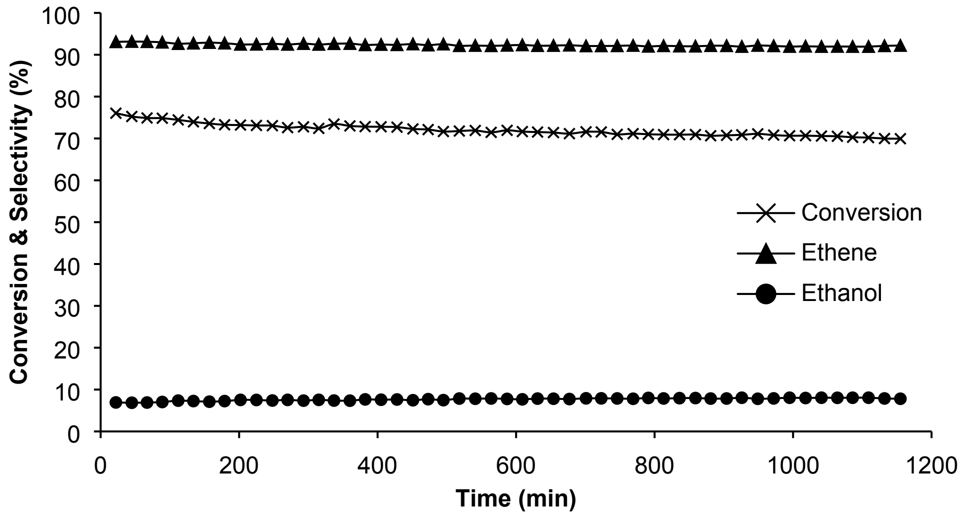

Figure S8. Long term stability of DEE elimination over 17%HPW/SiO_2 (0.20 g catalyst, 200 °C, 12 kPa DEE partial pressure, 20 ml min^{-1} N_2 flow rate).

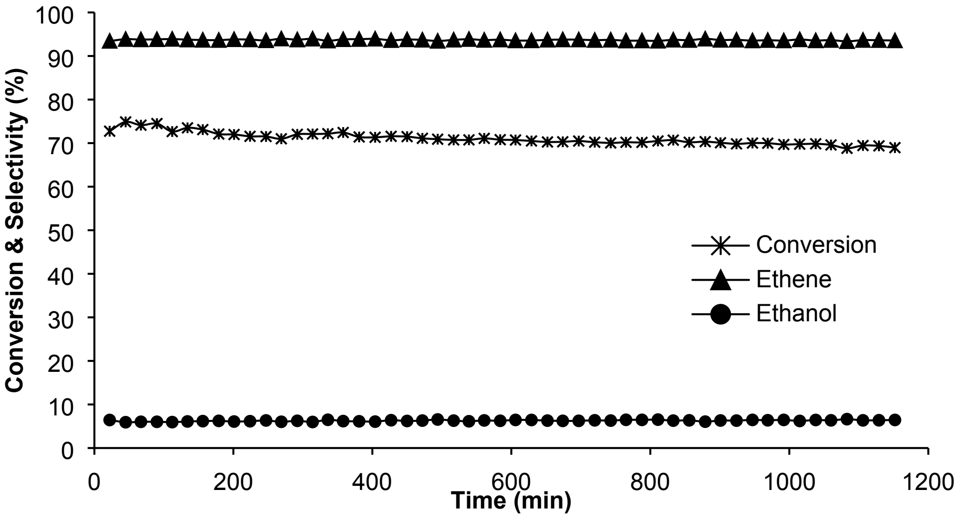

Figure S9. Long term stability of DEE elimination over CsPW (0.20 g catalyst, 200 °C, 12 kPa DEE partial pressure, 20 ml min^{-1} N_2 flow rate).

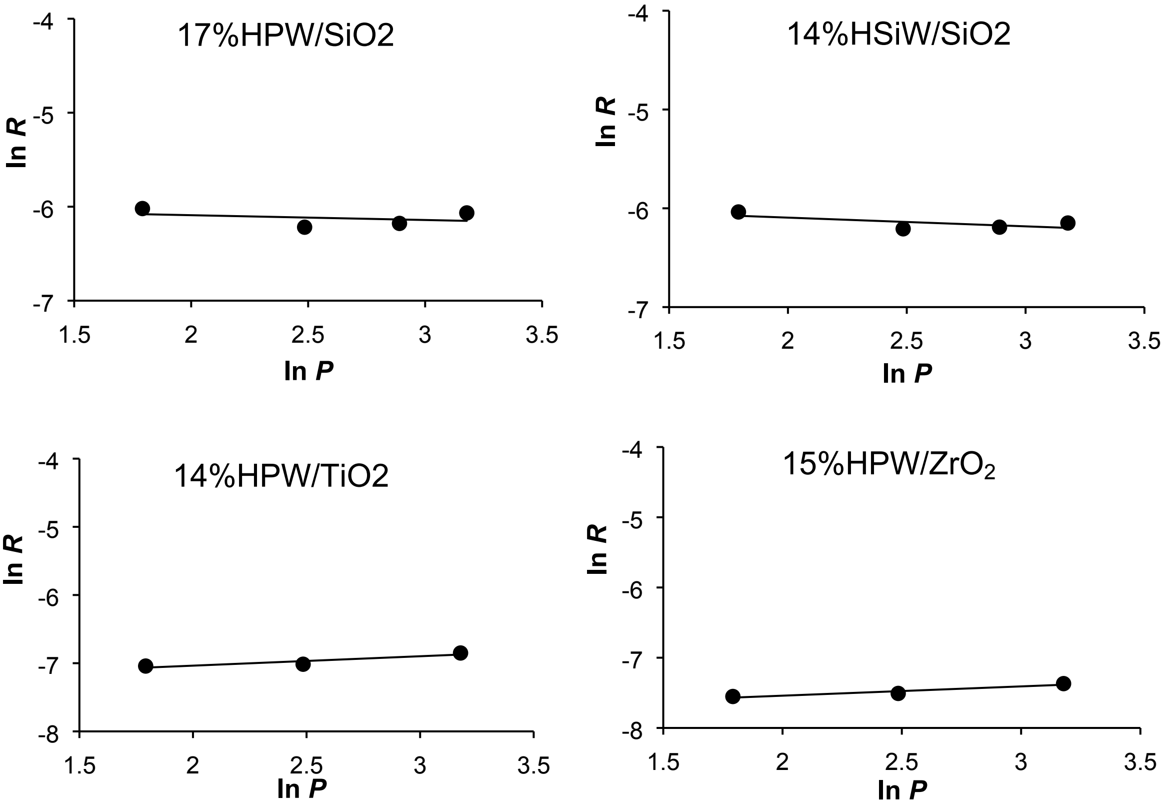

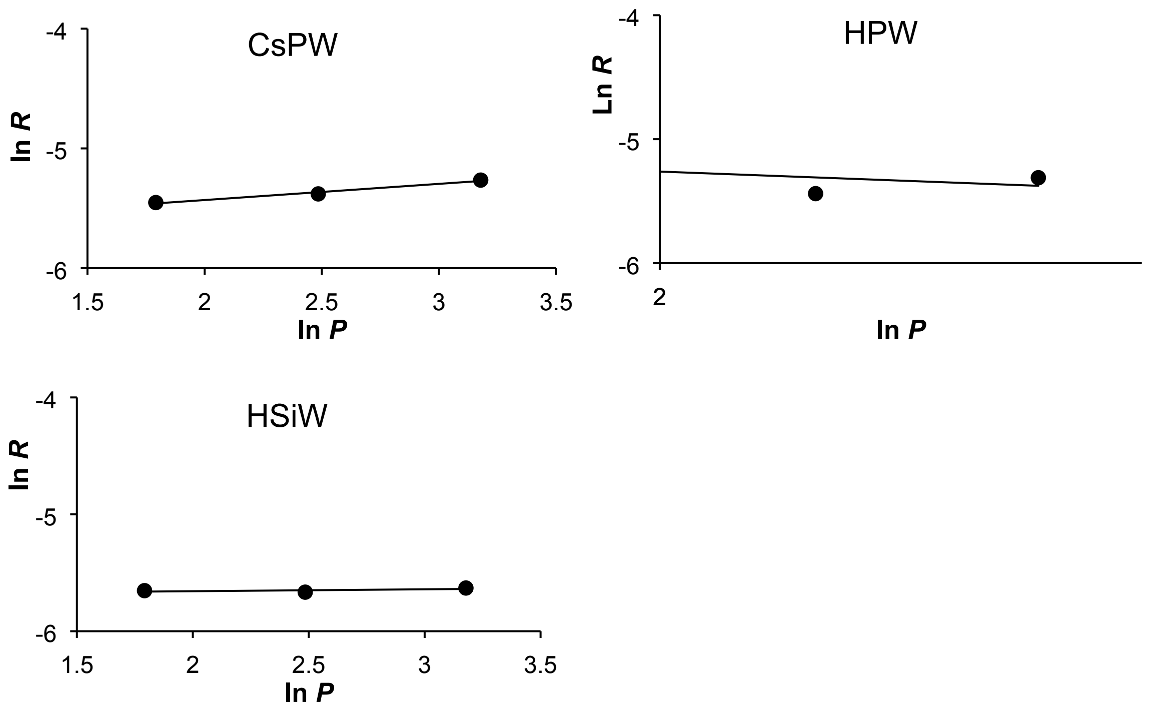

Figure S10. Logarithmic plot of reaction rate R (mol g^{-1}h^{-1}) vs. DEE partial pressure P (kPa) for DEE elimination at 150 ^{o}C and 20 ml min^{-1} flow rate over: 17%HPW/SiO_2 (0.20 g), 14%HSiW/SiO_2 (0.20 g), 14%HPW/TiO_2 (0.20 g), 15%HPW/ZrO_2 (0.20 g), CsPW (0.20 g), HPW (0.30 g) and HSiW (0.30 g).

Weisz–Prater analysis

The Weisz–Prater criterion, C_{WP} (eq. (4)), uses measured values of reaction rate to determine if internal diffusion is limiting the reaction.[2] If $C_{WP} \ll 1$, there are no diffusion limitations; when $C_{WP} \gg 1$, internal diffusion strongly limits the reaction.

$$C_{WP} = \frac{r\rho l^2}{DC_b} \quad (4)$$

In this equation, r is the observed reaction rate per unit catalyst weight (mol g^{-1}s^{-1}), ρ is the bulk density of catalyst (g cm^{-3}), l is the radius of catalyst particle (cm), D is the effective diffusion coefficient (cm^{2}s^{-1}) and C_b is the bulk concentration of substrate in the feed (mol cm^{-3}).

For DEE elimination on 17%HPW/SiO_2 at 150 °C and 12 kPa DEE partial pressure, the rate $r = XF/W = 1.99 \cdot 10^{-3}$ mol g^{-1}h^{-1} = $5.53 \cdot 10^{-7}$ mol g^{-1}s^{-1}(Figure 8 in the main text). Other parameters are as follows: $\rho = 0.39$ g cm^{-3},[1] $l = 0.56 \cdot 10^{-2}$ cm (average particle radius for the catalyst with 45 – 180 μm diameter of catalyst particles) and $C_b = 3.5 \cdot 10^{-6}$ mol cm^{-3} at 150 °C. The catalyst had a pore diameter of 15.8 nm in mesoporous range (Table 1). The Knudsen diffusion coefficient D for transport of DEE molecules into the pore was calculated using Equation (5):[3]

$$D = \left(\frac{d}{3}\right)\left(\frac{8RT}{\pi M}\right)^{1/2} \qquad (5)$$

where R is the gas constant (8.314 J K^{-1}mol^{-1}), T is the temperature (423.15 K), M is the molecular mass of DEE (0.074 kg mol^{-1}) and d is the mean pore diameter ($15.8 \cdot 10^{-9}$ m). From this equation, $D = 1.8 \cdot 10^{-7}$ m^{2}s^{-1} = $1.8 \cdot 10^{-2}$ cm^{2}s^{-1}.

Overall, from Equation (1), $C_{WP} = 1.1 \cdot 10^{-4} << 1$, which indicates no internal diffusion limitations.

References

(1) Al-Faze, R.; Finch, A.; Kozhevnikova, E. F.; Kozhevnikov, I. V. Dehydration of methanol and ethanol over silica-supported heteropoly acids in the gas phase: surface-type versus bulk-type catalysis mechanism. *Appl. Catal. A* **2020,** *597,* 117549.

(2) Weisz, P. B.; Prater, C. D. Interpretation of measurements in experimental catalysis. *Adv. Catal.* **1954,** *6*, 143–196.

(3) King, M. B.; Winterbottom, J. M. Kinetics of homogeneous reactions and of reactions on solid catalyst surfaces. In Reactor Design for Chemical Engineers, Winterbottom, J. N.; King, M. B., Ed.; Stanley Thorns: Padstow, 1999; p.154.
